# Pedigree-Based Deciphering of Genome-Wide Conserved Patterns in an Elite Potato Parental Line

**DOI:** 10.3389/fpls.2018.00690

**Published:** 2018-05-23

**Authors:** Xiaochuan Li, Jianfei Xu, Shaoguang Duan, Chunsong Bian, Jun Hu, Huolin Shen, Guangcun Li, Liping Jin

**Affiliations:** ^1^Institute of Vegetables and Flowers, Chinese Academy of Agricultural Sciences/Key Laboratory of Biology and Genetic Improvement of Tuber and Root Crops, Ministry of Agriculture, Beijing, China; ^2^College of Horticulture, China Agricultural University, Beijing, China; ^3^Bijie Institute of Agricultural Sciences, Bijie, China

**Keywords:** autotetraploid potato, elite parent, pedigree, reduced-representation resequencing, conserved segment

## Abstract

Elite parental lines are more likely to breed fine varieties, but knowledge about elite parents and their genetic backgrounds is limited. In this paper, we investigated the pedigree relationships of potato varieties bred worldwide and in China. Several elite parents were identified, and these parents were more frequently used as parents in breeding programs across different time periods and countries. We next used 2b-RAD, a reduced-representation sequencing method, to genotype the elite parent Mira and 24 of its offspring. These cultivars span 5 generations, making this lineage the longest continuous pedigree among Chinese bred potatoes. A total of 47,314 tetraploid single nucleotide polymorphisms (SNPs) identified by FreeBayes were used to trace the conserved segments of the Mira genome. The conserved segments had identical or similar allele-specific SNPs across the analyzed genotypes. In Mira, 3,788 segments comprising over 10,000 bp, or 20.8% of the genome, were defined as conserved segments. These segments contain genes involved in crucial biological processes that are of special interest to breeders. These regions, which have been conserved across generations of highly selective breeding, may be helpful for further breeding and performing genome-wide breeding by design.

## Introduction

Potatoes (*Solanum tuberosum* L.) were first cultivated in China between 1573 and 1620 (Sun, 2003) and then spread gradually to the entire country. A large number of varieties were introduced from abroad and some of these evolved into farm varieties (also called indigenous) during this process. Some of these farm varieties are still grown in China (Sun, 2003). The modern potato breeding program in China began in the 1930s, starting with the introduction of varieties such as Katahdin, Epoka, Mira, Anemone, and Schwalbe as well as the varieties selected from imported lines such as Xiaoyezi and Duozibai, to launch crossbreeding programs around the country. Chinese potato breeding programs are also cooperative with institutions around the world. In recent decades, over 600 varieties have been bred, and the Chinese-bred variety Kexin 1 is planted on nearly 0.8 million ha every year (Duan, 2013).

Elite parents are more likely to breed fine varieties in crossbreeding (Zhuang, 2003). The elite Chinese parents of Chinese potato varieties, Katahdin, Epoka, Mira, Anemone, Xiaoyezi, and Duozibai, were used to breed 74 validation varieties (68.8% of all validation varieties) before 1983 and 156 validation varieties (45% of all validation varieties) from 1983 to 2005 (Jin, 2007). Worldwide, there has been no comprehensive investigation of the elite potato parents in the breeding process. Mira, which was bred in 1952 in Germany and introduced into China during the 1950s, has been widely planted in southwest China and has used as a control/check during the breeding process (Duan, 2013). Its progenies, including Kexin 2, Chuanyu 10, and Zhengshu 5, are also widely planted in China, and Kexin 2 and Gaoyuan 7 are also frequently used as parents. Most significantly, the Mira lineage has the longest continuous pedigree among Chinese bred potatoes, with a clear breeding history spanning 5 generations, making Mira and its progeny ideal candidates for dissecting the conserved genome segments of elite potato parents.

Modern potato breeding is a highly artificial selective process, and usually only one variety is obtained from 200,000 crossing progenies (Sun, 2003). Breeding selects for specific alleles, which may contain key genes affecting traits of special interest (Peleman and van der Voort, 2003; Chen et al., 2013; Li and Zhang, 2013). Alleles in a chromosomal segment are marked with similar features of polymorphisms. Conserved chromosomal segments are usually characterized by reduced numbers of SNPs or by the presence of identical or similar allele-specific SNPs (Lai et al., 2010; Zhou et al., 2016). Variable genome segments are typically characterized by different allele-specific SNPs. The tendency of alleles in genomic segments to be conserved or variable becomes apparent after several generations of breeding (Yamamoto et al., 2010). So, tracing the origin of conserved chromosomal segments, which likely contain key genes that are of special interest to breeders, based on the pedigree relationships of parents and offspring is considered important for genetic research of elite parents (Zhou et al., 2003; Lai et al., 2010; Jiao et al., 2012; Zhou et al., 2016). For example, by comparing the SNPs of 11 progenitors and 8 progeny of the elite rice parent Huanghuazhan, 26.22% of the Huanghuazhan genome was found to be strictly conserved (Zhou et al., 2016). In addition, 101 chromosomal blocks with reduced numbers of SNPs were identified in 6 maize elite inbred lines, and a number of genes known to be under selection were found in these regions (Lai et al., 2010). Tracing the conserved chromosomal segments is usually done by identifying blocks of SNPs in the whole genome with the help of high-throughput sequencing technology.

In the last decade, high-throughput sequencing technology has greatly improved understanding of crop genetics, especially with the discovery of SNPs across the genome. For example, the potato reference genome, which was obtained by sequencing a homozygous doubled-monoploid potato clone (The Potato Genome Sequencing Consortium [PGSC], 2011) is important for genotyping. However, most potato cultivars are highly heterozygous autotetraploids (2*n* = 4× = 48). Genome assembly after resequencing autotetraploids, which is a precondition for genotyping, is a huge challenge, and no autotetraploid assembly has yet been obtained (The Potato Genome Sequencing Consortium [PGSC], 2011). Reduced-representation genotyping methods have been applied successfully in autotetraploids including alfalfa and potato (Uitdewilligen et al., 2013; Yu et al., 2016). With reduced-representation genotyping, fragments of the genome are selected for sequencing, but these methods differ in how genomic fragments are made and chosen (Rowan et al., 2017). For example, the genotype by sequencing (GBS) method, which has been previously been used to genotype potato, uses Adaptive Focused Acoustics to fragment genomic DNA to build a sequencing library (Uitdewilligen et al., 2013). In contrast, the 2b-RAD method is based on sequencing uniform fragments produced by type IIB restriction endonucleases and generates relative evenly distributed and highly reproducible SNPs with high density coverage in specific genomic regions (Wang et al., 2012). With the help of the package FreeBayes, which calls SNPs based on allele-specific read depths for sequences obtained by reduced-representation genotyping methods, SNPs are classified as nulliplex (*aaaa*), simplex (*aaab*), duplex (*aabb*), triplex (*abbb*), and quadruplex (*bbbb*) genotypes (Uitdewilligen et al., 2013).

In this paper, we used the 2b-RAD method to sequence the genome of the elite parent Mira and its 24 representative progenies after analyzing the pedigree of varieties in the potato pedigree database and varieties bred in China. Conserved chromosomal segments were revealed by analysis of pedigree relationships and SNP information. These regions, which contain key genes that affect traits of special interest to breeders, potentially provide *a priori* knowledge for the selection of favorable breeding parents and molecular breeding.

## Materials and Methods

### Potato Pedigree Investigation

The pedigree of potato varieties bred worldwide was investigated via the potato pedigree database^1^ (van Berloo et al., 2007). Pedigree information for Chinese-bred potatoes was collected from the validation reports of different varieties. The direct parents of all recorded cultivars were investigated, and the number of times a genotype was listed as a direct parent was determined. A direct parent was directly involved in crossing, as the father and/or mother of a variety. The recorded cultivars were classified in five categories: bred worldwide in 1841–2013, bred worldwide in 2003–2013, bred in China in 1930–2016, bred in China before 2000, and bred in China after 2000.

### Plant Materials and Genome Resequencing

Genomic DNA of Mira and its 24 offspring was isolated as described previously (van der Beek et al., 1992). The 2b-RAD reduced-representation sequencing strategy was used to generate libraries, which were sequenced by HiSeq X-Ten at Oebiotech, China (Wang et al., 2012). Diploid SNPs were called after aligning reads to the reference genome^2^ (The Potato Genome Sequencing Consortium [PGSC], 2011), DM1-3 516 R44 (hereafter referred to as DM), using SOAP2 software (parameters -M 4, -v 2, -r 0) as described by Fu et al. (2013). Tetraploid SNPs were called using the FreeBayes package as described previously with the filters minimum depth of 15× and minimum genotype quality (GQ) of 26 (Uitdewilligen et al., 2013). Variance analysis was performed using SPSS Statistics 22 (SPSS GmbH Software, Munich, Germany). The distribution of tags, SNPs, and genes was drawn using a Perl script with the GD module^3^.

Candidate sites were randomly selected for validation, including approximately equal numbers of heterozygous and homozygous SNP calls. Primers were designed using BatchPrimer3 based on the PSGC assembly to amplify a 200 to 400-bp fragment flanking each target site (**Supplementary Table S1**; You et al., 2008). PCR products were sequenced using the Sanger method to verify 2b-RAD genotypes. SNPs were called in a dosage-dependent manner in the sequence trace files as *aaaa* (nulliplex), *aaab* (simplex), *aabb* (duplex), *abbb* (triplex), or *bbbb* (quadruplex) using DAx software (Van Mierlo Software Consultancy, Eindhoven, Netherlands). The heterozygous groups *aaab*, *aabb*, and *abbb* were distinguished based on the relative peak height of each nucleotide in the sequence trace file.

### Conserved and Variable Chromosome Segments Detection

The conserved and variable segments were detected based on the inherited ratio of polymorphic sites from Mira to its progeny (if one site in Mira is *aaaa* and *aabb* in one of its offspring, the ratio is 0.5; if the site in another of its offspring is *aaaa*, the ratio is 1). Two strategies were employed. First, all progenies of Mira were assumed to have the same pedigree status, which ignores the fact that these progenies are from different generations. In practice, we calculated all the SNP inherited ratio as described above, and then averaged the ratio among progenies site by site. For the second strategy, pedigree status was taken into account. Mira and its progeny span 5 generations, which led to different minimum inherited ratio between generations. For instance, the minimum inherited ratio of polymorphic sites from Mira in first generation offspring is 0.5, and in second generation offspring the minimum ratio is 0.25. In actuality, we calculated inherited ratio for all direct progeny of Mira for all SNP sites, and then averaged the ratio site by site as described above. If one progeny also served as a parent, we also calculated the inherited ratio of its progeny and multiplied the ratio among generations for each site. Then we averaged the ratio among collateral lines. Ratios were calculated for both diploid and tetraploid SNPs. Frequency calculation, correlation analysis, and generation of the QQ plots were performed in SPSS Statistics 22 (SPSS GmbH Software, Munich, Germany). A diagram of chromosomal segments with different inherited ratio was drawn using matlab7 (MathWorks, Inc., Natick, MA, United States).

The borders of a segment were defined as the midpoints of adjacent polymorphic sites with different inherited ratio. The highly skewed tails of QQ plot for both minimum and maximum values of inherited ratio were analyzed for the conserved and variable segments. We first used the top 5% (inherited ratio > 0.973) as a cut-off to identify the conserved segments, but a very similar ratio of 0.96875 resulted in the inclusion of a large proportion (6.8%) of all segments; therefore, we decided to use 0.96875 as a threshold. Segments with inherited ratio > 0.96875 may be highly conserved and were named highly conserved regions. Segments with inherited ratio > 0.9 were named conserved regions. We used the inherited ratio < 0.306 (5% of all chromosome segments) as a cut-off for identification of the named variable segments. The genes in these regions were identified, and gene ontology (GO) enrichment analysis was performed as described previously (Goyer et al., 2015).

## Results

### Potato Pedigree Investigation Indicates the Existence of Core Elite Parents

An online potato pedigree database, developed by Wageningen University and consisting of over 8,000 genotypes bred in 68 countries, reflects most of the potato breeding history worldwide (van Berloo et al., 2007). A total of 4,397 cultivars with clear pedigree information and bred between 1841 and 2013 were investigated. The direct parents of these 4,397 cultivars and the number of times a cultivar was listed as a direct parent were investigated. Fifteen genotypes were listed as a direct parent ≥30 times (**Table 1**) and were used to breed 655 out of 4,397 (14.9%) cultivars (**Table 2** and **Supplementary Table S2**), and 295 genotypes were listed as a direct parent ≥5 times (**Table 2** and **Supplementary Table S2**). Katahdin, which was bred in 1932 in the United States and has 97 direct progenies in the potato pedigree database, was the most frequently used parent worldwide between 1841 and 2013. The ancestors of Katahdin include Early Rose, Erste von Fromsdorf, Paterson’s Victoria, Rural New Yorker 2, and Sutton’s Flourball. The direct progenies of Katahdin include Houma, Wauseon, Sebago, and Pontiac; the offspring of Katahdin include Shepody, Atlantic, Innovator, Desiree, Favorita, Spunta, and Maris Piper. These results indicate that some genotypes were used as parents more frequently in potato breeding.

**Table 1:** The 10 cultivars most frequently used as parents of worldwide-bred potato varieties.

10 most common direct parents of potato varieties bred worldwide			
**Released potato varieties in 1841–2013**		**Released potato varieties in 2003–2013**	
**Direct parent**	**Times used as parents**	**Direct parent**	**Times used as parents**
Katahdin	97	Agria	27
Agria	62	Innovator	13
Desiree	62	Valor	12
Aquila	48	Felsina	11
Jubel	42	Victoria	11
Maris Piper	42	Marabel	9
Flava	40	Seresta	9
Early Rose	39	Laura	8
Cara	38	Mondial	8
Am66-42	33	Nicola	8

**Table 2:** Statistics for preferred parents in the potato pedigree study.

	Released potato varieties worldwide in 1841–2013	Released potato varieties worldwide in recent 10 years (2003–2013)	Chinese bred potato varieties	Chinese bred potato varieties before 2000	China bred potato varieties after 2000
No. of total varieties	4397	510	523	193	330
					
No. of genotypes as direct parent ≥10 times	15 (≥30 times)	5	8	5	2
No. of varieties bred by these parents	655	76	143	89	22
Ratio of varieties bred by these parents	14.90%	14.90%	27,34%	46.11%	6.67%
					
No. of genotypes as direct parent ≥3 times	295 (≥5 times)	63	81	25	57
No. of varieties bred by these parents	2524	261	328	127	190
Ratio of varieties bred by these parents	57.40%	51.18%	62.72%	65.80%	57.58%
Times as male/female/both parents of these parents	1619/1689/765	158/174/65	226/239/129	78/104/55	120/139/69

To more specifically reflect the modern breeding program, 510 cultivars bred worldwide between 2003 and 2013 were investigated. Five genotypes, which were listed as a direct parent >10 times (**Table 1**), were used to bred 76 out of 510 (14.9%) cultivars (**Table 2** and **Supplementary Table S2**), and 63 genotypes were listed as a direct parent ≥3 times (**Table 2** and **Supplementary Table S2**). Agria, which has 27 direct progenies, was one of the most frequently used parents for potato breeding from 2003 to 2013 and has 62 direct progenies among all 4,397 cultivars developed between 1841 and 2013. Agria was bred in Germany in 1985, and its ancestors include Industrie, Jubel, Binjie, Jaune d’Or, and Clivia, which are quite different from the ancestors of Katahdin’s ancestors. These results indicate that the parents preferred in breeding changed over time.

Because the Chinese bred potato varieties were not included in the potato pedigree database, we collected cultivar validation information up to 2016, and 523 bred cultivars with detailed information were used for pedigree investigation. Eight genotypes, which were listed as a direct parent >10 times (**Table 3**), were used to breed 143 out of 523 (27.34%) cultivars (**Table 2** and **Supplementary Table S2**), and 81 genotypes were listed as direct parents ≥3 times (**Table 2** and **Supplementary Table S2**). To determine the changes in preferred parents during different periods, we further divided these 523 Chinese cultivars into two groups: cultivars bred before 2000 and those bred after 2000. Before 2000, 193 cultivars and 5 genotypes, which were listed as the direct parent >10 times (**Table 3**), were used to breed 89 out of 193 (46.11%) cultivars (**Table 2** and **Supplementary Table S2**), and 25 genotypes were listed as direct parents ≥3 times (**Table 2** and **Supplementary Table S2**). After 2000, 330 cultivars and only 2 genotypes were listed as a direct parent >10 times (**Table 3**), and 57 genotypes were listed as the direct parent ≥3 times (**Table 2** and **Supplementary Table S2**). These results indicate that some genotypes were used as parents more frequently in potato breeding and that more germplasm was used in potato breeding after 2000.

**Table 3:** The 10 cultivars most frequently used as parents of Chinese-bred potato varieties.

10 most common direct parents of Chinese bred potato varieties					
**Chinese bred potato varieties (1940–2016)**		**Chinese bred potato varieties before 2000**		**Chinese bred potato varieties after 2000**	
**Direct parent**	**Times**	**Direct parent**	**Times**	**Direct parent**	**Times**
Duozibai	31	Duozibai	31	Neishu 7	11
Epoka	20	Epoka	17	Favorita	11
Schwalbe	20	Katahdin	16	Shepody	9
Katahdin	19	Schwalbe	13	Zaodabai	8
Mira	14	Mira	10	Kexin 2	7
Kexin 2	13	Anemone	9	Schwalbe	7
Favorita	11	Xiaoyezi	9	Atlantic	7
Neishu 7	11	Kexin 2	6	Zhongshu 3	7
Shepody	9	Zishanyao	6	Cooperation 23	6
Xiaoyezi	9	Gaoyuan 7	5	Qinyu 30	6

**FIGURE 1:**
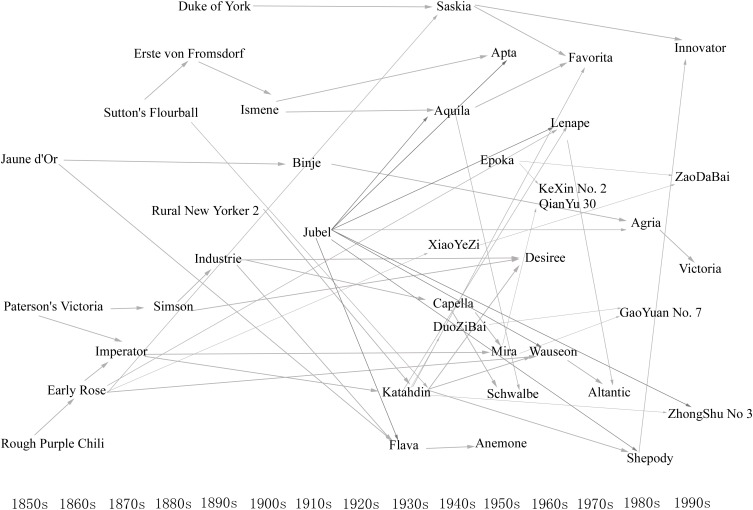
Relationships between elite parents. Elite parents are arranged based on year bred. Arrows indicate the pedigree relationships between elite parents, but do not indicate direct relationships.

We further investigated the pedigree relationships of the elite parents. In the 1850s, the first generation of elite parents, such as Rough Purple Chili, Jaune d’Or, and Paterson’s Victoria, appeared in the potato pedigree database. These parents were crossed with each other or with other parents to gradually form a new generation of elite parents, and this process was repeated. Several elite parents in early generations included Early Rose, Jubel, and Industrie. From the 1930s–1950s, China imported elite parents such as Katahdin, Epoka, Mira, Anemone, and Schwalbe, and the elite parents selected from imported lines, such as Xiaoyezi and Duozibai. In later years, some of the offspring of these parents also served as elite parents, such as Kexin 2, Gaoyuan 7, Zaodabai, and Zhongshu 3 (**Figure 1**). This indicates that excellent parents appeared in different periods; some of the offspring of elite parents were also excellent parents, and some of elite parents shared the same ancestors, implying that some genes were inherited during artificial selection. So we can infer that the ideal models for studying the conserved genome segments of elite parents are the excellent parents that appeared in different generations of the same family.

### Genotyping of the Mira Family

**FIGURE 2:**
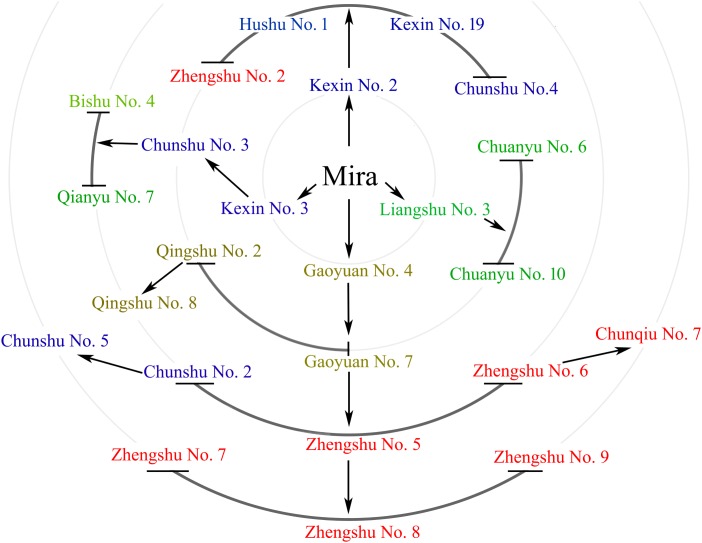
Mira and 24 of its offspring. Mira and 24 of its offspring are shown in different circles that indicate different generations. Arrows indicate direct relationships.

**FIGURE 3:**
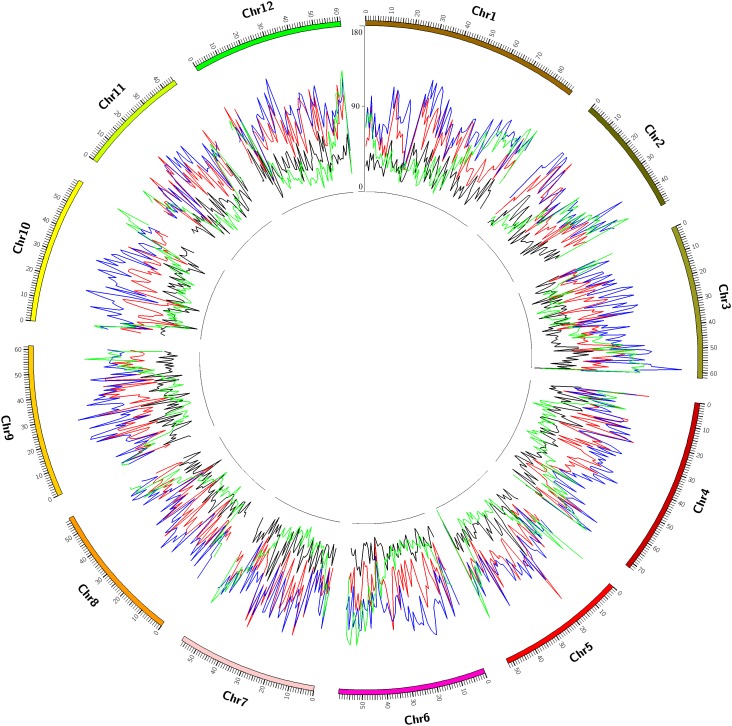
A dense map of tags, diploid single nucleotide polymorphisms (SNPs), tetraploid SNPs and genes. A dense map of unique tags (blue lines), diploid SNPs (red lines), tetraploid SNPs (yellow lines), and genes (green lines) per 1M window 1 Mb slides of the potato genome.

We sequenced the genomes of Mira and 24 representative progenies from different generations (**Figure 2**). Progeny derived from Mira, such as Kexin 2 and Gaoyuan 7, have also served as excellent parents. The Mira lineage spans 5 generations and is the longest continuous pedigree in China, making Mira and its progeny ideal candidates for dissecting the conserved genome segments of elite parents. The reduced-representation genotyping method, 2b-RAD, was used to generate libraries for sequencing on the HiSeq X-Ten platform (Fu et al., 2013). After aligning 48,219,922 reads to the DM reference genome, 120,460 unique tags were obtained, and the average sequencing depth was 124×. The average number of tags per 1M genome was 90.38 (σ = 19.26) (**Figure 3** and **Table 4**). In these tags, 113,157 sites showed SNPs. The number of alleles unique to a cultivar ranged from 0 to 2,433, and in general the parents had fewer unique alleles (**Table 4**). A total of 104,732 diploid SNPs were identified using SOAP2 (an average of 71.38 SNPs per 1M genome, σ = 20.57) (**Figure 3** and **Table 4**), and 52,239 tetraploid SNPs were identified using FreeBayes (an average of 37.1 SNPs per 1M genome, σ = 12.46) (**Figure 3** and **Table 4**). A total of 3,066 diploid SNPs and 1,781 tetraploid SNPs exhibited polymorphism against the reference genome but were not polymorphic among samples. We randomly selected 220 tetraploid SNPs identified in the samples for Sanger sequencing to validate the FreeBayes genotyping, and 93.19% SNPs had the correct genotype. The longest distance between diploid SNPs was 315,022 bp, with an average distance of 7,110 bp, and the longest distance between tetraploid SNPs was 483,592 bp, with an average distance of 13,769 bp. In potato, the LD decay value is at least 600 Kb and between 2 and 4 Mb on average; therefore, the SNP density was sufficient for our study (Simko et al., 2004; D’hoop et al., 2010; Vos et al., 2017).

**Table 4:** Single nucleotide polymorphism identification in the Mira family of potato.

	Diploid SNPs	Tetraploid SNP
SNPs in all samples	104,732	52,239
		
SNPs per sample	40,845–49,533	12,527–19,417
(mean)	45,174	15,737
		
SNPs of no polymorphism among samples	3,066	1,781
		
SNPs per 1M genome	12–152	3–94
(mean)	71.38	37.1
		
Longest distance between SNPs	1–315,022	1–483,592
(mean)	7,110	13,769
		
Alleles of cultivar-unique per samples	0–2,433	
(mean)	521	

### Inherited Ratio in the Mira Family

**FIGURE 4:**
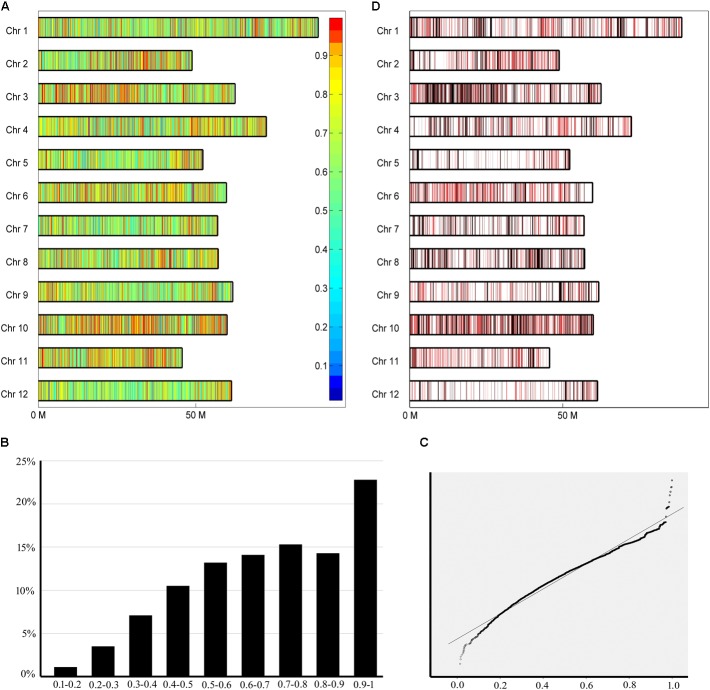
Chromosome segments with different inherited ratio. **(A)** Different inherited segments in the Mira genome. Borders of a segment are defined as the midpoints of adjacent polymorphic sites with different inherited ratio. Inherited ratio are shown in different colors as indicated in the legend to the right of the figure. **(B)** Histogram showing the distribution of inherited ratio. The proportion of SNPs with different inherited ratio is shown. **(C)** QQ plot showing the distribution of inherited ratio. Inherited ratio < 0.5 and >0.95 are highly skewed from the expected values. **(D)** Distribution of highly conserved segments (red) and conserved segments (black).

To trace the conserved chromosome segments, the inherited ratio were calculated based on information about SNP sites and pedigree relationships between Mira and its progenies. Because the progenies were selected from different generations, the pedigree relationships between different generations were also considered. All tetraploid SNPs and diploid SNPs were separately used to calculate the inherited ratio (**Figure 4A** and **Supplementary Figure S1**). We found that although the ratio have a distribution close to normal, ratio were not normally distributed based on the Kolmogorov–Smirnov test (**Figure 4B** and **Supplementary Figure S2**). QQ plot was used to identify which inherited ratio deviated from a normal distribution (Filliben, 1975). The highly skewed tails (ratio < 0.05 and >0.95) implied that selection was exerted during reproduction, since if there is no selective influence, the ratio should be normally distributed (**Figure 4C** and **Supplementary Figure S3**). The inherited ratio of tetraploid SNPs and diploid SNPs at the same sites were significantly correlated at the 0.01 level (*r* = 0.763). There is a small difference in SNP inherited ratio because the *ab* heterozygous polymorphism sites in diploid SNPs became *abbb*, *aabb*, and *aaab* in tetraploid SNPs. We also calculated the ratio while assuming all progenies had the same pedigree status (**Supplementary Figure S4**). We found that the highly conserved sites (inherited ratio > 0.96875) identified by this method included the sites identified when considering pedigree status (significantly correlated at 0.01 level, *r* = 0.69). The number of highly conserved sites was reduced drastically when we considered pedigree status. This is largely because the minimum rate of transfer of parent DNA polymorphisms to different generations of offspring is different, and these differences are ignored when assuming all progenies have the same pedigree status.

### Investigation of the Conserved Chromosome Segments

During artificial selection on potato, different alleles contained in parental chromosome segments may have different fates, including conservation or variation because the resulting traits do or do not meet the needs of breeders (Chen et al., 2013; Li and Zhang, 2013). The tendency of alleles in chromosomal segments to be conserved or variable may be more apparent after several generations of breeding (Yamamoto et al., 2010). In our study, we determined whether a chromosome segment is conserved based on the inherited ratio (see above). The borders of a segment were defined as the midpoints of adjacent polymorphic sites. A total of 43,412 segments with different inherited ratio were obtained. The highly conserved segments with inherited ratio > 0.96875 reflected the highly skewed tail from the distribution of inherited ratio (**Figure 4C**). These chromosome segments accounted for 11.8% of the Mira genome and included 2,575 chromosome segments with sizes over 10,000 bp. The longest segment was 358,489 bp, with segments 31,507 bp on average. There were 97 to 408 highly conserved segments distributed on each chromosome (**Figure 4D** in red). An average of 1.84 genes was included in each highly conserved segment. The conserved segments with inherited ratio > 0.9 accounted for 20.8% of the Mira genome and included 4,037 chromosome segments with sizes > 10,000 bp. The longest segment was 358,489 bp, with an average segment length of 35,692 bp. There were 175 to 509 conserved segments distributed on each chromosome (**Figure 4D** in black). A 36.49 to 130.93% increase in the number of conserved segments was observed when 0.9 was used as a threshold compared with 0.96875. An average of 1.94 genes was included in each conserved segment. These results indicate that some parts of the Mira genome remained highly conserved after several generations of selection. Lines are selected during breeding if they have the traits of interest, thus the conserved chromosome segments may contain genes that are of interest to breeders.

**FIGURE 5:**
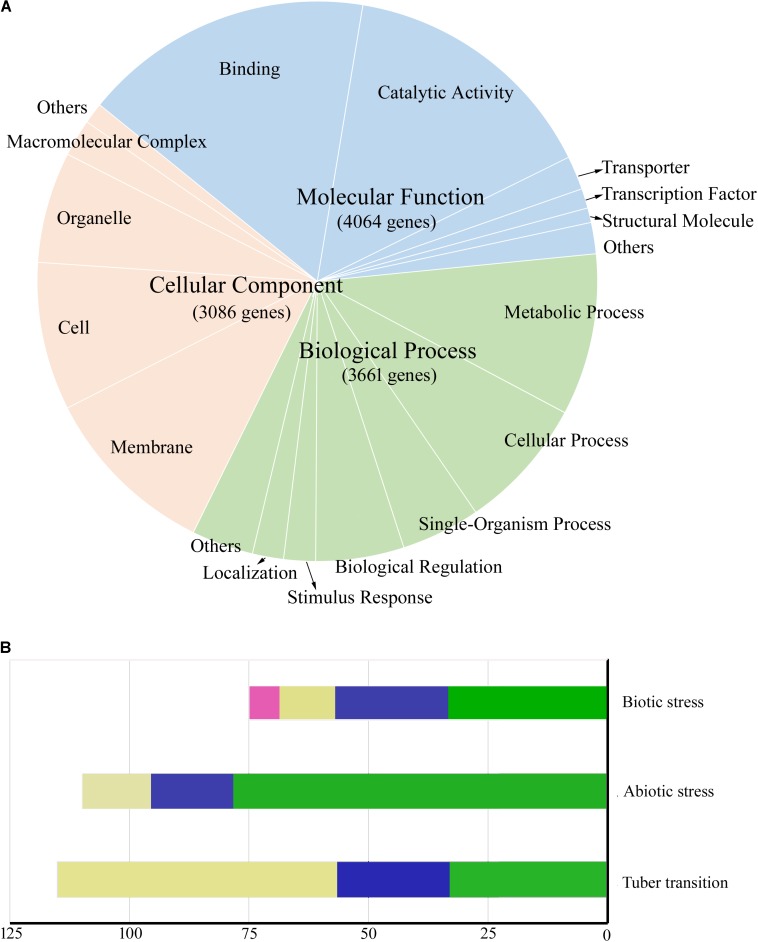
Functions and expression patterns of genes located in conserved segments. **(A)** Pie chart showing the proportion of genes located in conserved segments that are annotated with different Cellular Component (orange), Molecular Function (blue), and Biological Process (green) gene ontology terms. **(B)** The number of genes differentially expressed during biotic stress, abiotic stress, and tuber formation (10-fold). Biotic stress samples include wounded leaves (green), acibenzolar-s-methyl (BTH)-treated leaves (blue), DL-b-amino-n-butyric acid (BABA)-treated leaves (yellow), and *Phytophthora infestans*-infected leaves (red). Abiotic stress conditions include 35°C heat (green), mannitol stress (blue), and salt stress (yellow). The number of genes differentially expressed during the tuber transition include genes upregulated (10-fold) during the stolen to young tuber transition (green) and the young tuber to mature tuber transition (blue), and genes downregulated (10-fold) during the young tuber to mature tuber transition (yellow).

Gene ontology was used to predict the functions of genes contained in the conserved chromosome segments, and 4,043 and 6,538 genes in highly conserved and conserved segments had GO annotations, respectively. A total of 5,484 genes in conserved segments were annotated with terms from the categories: molecular function, biological process, and cellular component, and some were predicted to be involved in crucial biological processes, such as sugar metabolic process, photosynthesis, transport and signal transduction (**Figure 5A**). Genes with similar functions were not concentrated in a specific region or clustered together. The transcripts of 7,632 genes in conserved segments were found in DM or RH89-039-16 (hereafter referred to as RH) samples in PGSC (The Potato Genome Sequencing Consortium [PGSC], 2011), including 5,027 in leaves and 5,029 in tubers (4,392 in both). During the transition from stolen to tuber, 38 genes were highly expressed, and during the transition from young tubers to mature tubers 21 genes were upregulated and 56 genes were downregulated (>10-fold) (**Figure 5B**). We also found that 181 genes were highly expressed under biotic and abiotic stress (>10-fold) (**Figure 5B**). Furthermore, we found some characterized functional genes in these regions that are of interest to breeders (**Table 5**). Several genes related to photosynthesis were located in conserved segments, such as the plastidial Calvin cycle protein CP12, which regulates Calvin-Benson cycle enzymes (Graciet et al., 2004), Plastidial phosphoglucoisomerase 1 (*PGI1*), which provides phosphate sugar metabolites for the transient accumulation of starch in photosynthetic leaves (Geigenberger, 2011), and Transaldolase (*Tal1*). Other genes involved in carbohydrate metabolism and transport were also found in conserved segments, including, α-glucosidase (*Agl*), disproportionating enzyme (*Dpe-P*), starch synthase III (*SSIII*), UDP-glucose pyrophosphorylase (*UGPase*), pyrophosphate-fructose-6-phosphate α subunit (*Pfp-α*), sucrose synthase 3 (*Sus3*), adenylate transporter (*Ant*), plasma membrane H+-ATPase (*Pha1*), pentose-5-phosphate 3-epimerase (*Ppe*), inorganic phosphate transporter 1(*Pt1*), Enolase (*Eno*), and nucleoside diphosphate kinase (*Ndpk*) (Chen et al., 2001; Carpenter et al., 2015; Schönhals et al., 2016; Van Harsselaar et al., 2017).

**Table 5:** Characterized genes of special interest to breeders located in conserved segments.

Gene	Inherited ratio	Gene function	Reference	Verification test in reference
*PHYTOCHROME B*	0.97	Tuber induction	Jackson et al., 1998	The photoperiodic control of tuberization in silenced *PHYB* plants was abolished
*CONSTANS*	0.98	Tuber induction	Martinez-Garcia et al., 2002	*AtCO* overexpression impairs tuberizations but not alter photoperiod perception
*STBEL5*	0.96	Tuber induction	Chen et al., 2003	Mobile signals from leaf to stolon, overexpressed StBEL5 exhibited enhanced tuber formation even under non-inductive conditions
*Rca*	0.93	CO_2_ fixation	Li et al., 2010	Affect tuber starch content and starch yield in association mapping
*Pain1*	0.98	Vacuolar invertase	Li et al., 2010	Affect tuber chip color, starch content and starch yield in association mapping
*AuxRP*	0.93	Auxin-regulated protein	Sołtys-Kalina et al., 2015	Affect tuber chip color in a population (DG 97-952 and DG 08-26/39)
*InvGE*	0.92	Apoplastic invertase	Duarte-Delgado et al., 2017	Affect tuber chip color, sucrose content in association mapping
*GBSSI,wx*	0.97	Starch biosynthesis	van de Wal et al., 2001	Affect tuber starch composition in correlation test
*SSIII*	0.93	Starch C3 phosphorylation	Carpenter et al., 2015	Affect tuber starch C3 phosphorylation in association mapping

### Investigation of the Variable Chromosome Segments

In contrast to the conserved chromosome segments, which did not change over time, the variable chromosome segments varied after several generations of breeding. These variable chromosomal segments may have contained alleles, which coded for traits not favorite to breeding, and those segments will probably require improvement in the genome of the elite parent line Mira. 5% of chromosome segments with inherited ratio < 0.306 were defined as variable chromosome segments, and reflected the lower side of the highly skewed tail from the distribution of inherited ratio (**Figure 4C**). 1142 variance chromosome segments with sizes over 10,000 bp were found in the genome, and 54 to 147 segments were distributed on each chromosome (**Supplementary Figure S5**). A total of 1328 genes with annotated functions were found in the variance chromosomal segments, and 1122 of these genes were annotated as belonging to the categories of molecular function, biological processes, or cellular components. Transcripts of the 2,150 genes in variable segments were found in DM or RH samples in PGSC (The Potato Genome Sequencing Consortium [PGSC], 2011). Eight genes were highly (>10-fold) expressed during the transition of tubers, 9 genes were highly expressed under biotic stress, and 33 were highly expressed under abiotic stress. Furthermore, the characterized functional genes included *G6pdh (Glucose-6-phosphate dehydrogenase)*, which is associated with the trait of chip quality, and *Gap C (Glyceraldehyde 3-phosphate Dehydrogenase)*, and *GMPase (GDP-mannose Pyrophosphorylase)*, which are involved in carbohydrate metabolism (Chen et al., 2001; Li et al., 2008).

## Discussion

### Elite Parents Are Frequently Used in Potato Breeding

In this paper, we investigated the pedigree relationships of potatoes cultivated worldwide and those bred in China (van Berloo et al., 2007). We found that elite parents make up a large proportion of the varieties used as direct parents during breeding. Of the 4,397 cultivars cultivated worldwide, 14.9% were bred from 15 genotypes, and of 523 Chinese cultivars, 27.34% were bred from 8 genotypes. During different periods, the preferred parents changed (**Tables 1–3**). New generations of elite parents were largely the progenies of earlier ones. In China, imported cultivars such as Katahdin, Epoka, Mira, Anemone, Schwalbe, Xiaoyezi, and Duozibai, and some of their offspring, such as Kexin 2, Gaoyuan 7, and Zaodabai, served as elite parents (**Figure 1** and **Table 2**). This implies that elite parents passed on genes that were selected during breeding.

### Certain Regions Are Conserved in the Potato Genome

In this study, we considered not only the challenges of assembling the highly heterozygous autotetraploid potato genome, but also the importance of obtaining an even distribution of polymorphic sites at a sufficient density given LD decay (**Figure 3**). Thus, we chose to use the reduced-representation genotyping method, 2b-RAD, to identify SNPs (Simko et al., 2004; D’hoop et al., 2010; The Potato Genome Sequencing Consortium [PGSC], 2011; Wang et al., 2012; Vos et al., 2017). The conserved segments were assessed based on the difference in tetraploid SNPs between Mira and its offspring (**Figures 4A,D**). The pedigree status of different generations of Mira offspring was considered when we calculated the inherited ratio instead of assuming all progeny had the same pedigree status (**Supplementary Figures S3**, **S4**). We did not utilize the common sequence diversity parameters of Watterson’s 𝜃, Tajima’s π, or Tajima’s D because we sequenced only a small portion of the genome, and this sequencing method generated SNPs equally distributed across the genome (Waterson, 1975; Tajima, 1989; Wang et al., 2012). Moreover, we did not set a minimum segment size because it would potentially equalize the inherited ratio among different polymorphism sites in the set length segments. Based on inherited ratio thresholds of 0.96875 and 0.9, 11.8% and 20.8% of the Mira genome is conserved, respectively (**Figures 4B,C**). An elite parent line should demonstrate many traits that meet the needs of breeders. Genes we found in conserved segments were involved in many crucial biological processes, including characterized genes of special interest to breeders (**Figures 5A,B** and **Table 5**). These results are consistent with other studies in rice, and maize, in which conserved regions containing genes of interest to breeders were also distributed throughout the genome (Lai et al., 2010; Zhou et al., 2016). In contrast, the alleles found in variable segments of the Mira genome are not likely to be ideal, and these regions can be improved by use of other genotypes.

In this study, we sequenced only one family using a reduced-representation method. More detailed information will be obtained when additional elite genotypes are sequenced with advanced technology. When the genome conservation patterns of cultivated potato are established, other genomic regions could be possibly filled with the alleles of genes with crucial functions, such as disease, virus, pest, and drought resistance (Peleman and van der Voort, 2003; Li and Zhang, 2013). Moreover, cultivated potatoes are classified as different types (e.g., high-yield, starch-yield, processing potatoes), and the genome conservation patterns of these different types could be established after sequencing sufficient genotypes of the specific types (Hirsch et al., 2013). Cultivated potatoes make up only a small portion of the potato family and much more abundant germplasm resources that have desired traits exist in the so-called wild potatoes (Hijmans and Spooner, 2001; Rodríguez et al., 2010; Machida-Hirano, 2015). Cultivated potatoes are facing a shrinking genetic base (Hirsch et al., 2013). Breeders have invested a lot of energy and time into reversing this trend, for example by neo-tuberosum breeding and utilizing wild potatoes (Glendinning, 1976). Knowledge of genome conservation patterns will potentially accelerate the breeding process by defining which parts of the genome are conserved and which parts can be improved. Therefore, the established patterns of conservation aid the design of effective breeding platforms.

## Author Contributions

XL designed the study, conducted the experiments, performed the data analysis, and wrote the manuscript. JX designed the study and wrote the manuscript. SD and CB designed the study and participated in the data analysis. JH participated in the data analysis and wrote the manuscript. HS designed the study. GL designed the study, performed the data analysis, wrote the manuscript, and supervised the research. LJ designed and supervised the research. All authors have read and approved the final manuscript.

## Conflict of Interest Statement

The authors declare that the research was conducted in the absence of any commercial or financial relationships that could be construed as a potential conflict of interest.

## Footnotes

1 https://www.plantbreeding.wur.nl/PotatoPedigree/

2 http://solanaceae.plantbiology.msu.edu/pgsc_download.shtml

3 https://www.perl.org

## Supplementary Material

FIGURE S1 Different inherited segments in the Mira genome defined based on diploid single nucleotide polymorphisms (SNPs).

FIGURE S2 Histogram showing the distribution of inherited ratio calculated based on diploid SNPs.

FIGURE S3 QQ plot showing the distribution of inherited ratio calculated based on diploid SNPs.

FIGURE S4 Histogram showing the distribution of inherited ratio calculated assuming all progenies have the same pedigree status.

FIGURE S5 Distribution of highly variable segments.

TABLE S1 Primers used for single nucleotide polymorphism (SNP) validation.

TABLE S2 Preferred parents in potato breeding.

## Abbreviations

2b-RAD: restriction site-associated DNA
CIP: International Potato Center
DM: DM1-3 516 R44
LD: linkage disequilibrium
PGSC: Potato Genome Sequencing Consortium
RH: RH89-039-16
SNP: single nucleotide polymorphism
