# Supplementary figures and images for: Pedigree-Based Deciphering of Genome-Wide Conserved Patterns in an Elite Potato Parental Line
Source: Front Plant Sci. 2018 May 23;9:690. doi: 10.3389/fpls.2018.00690 (PMC5974212; doi:10.3389/fpls.2018.00690)

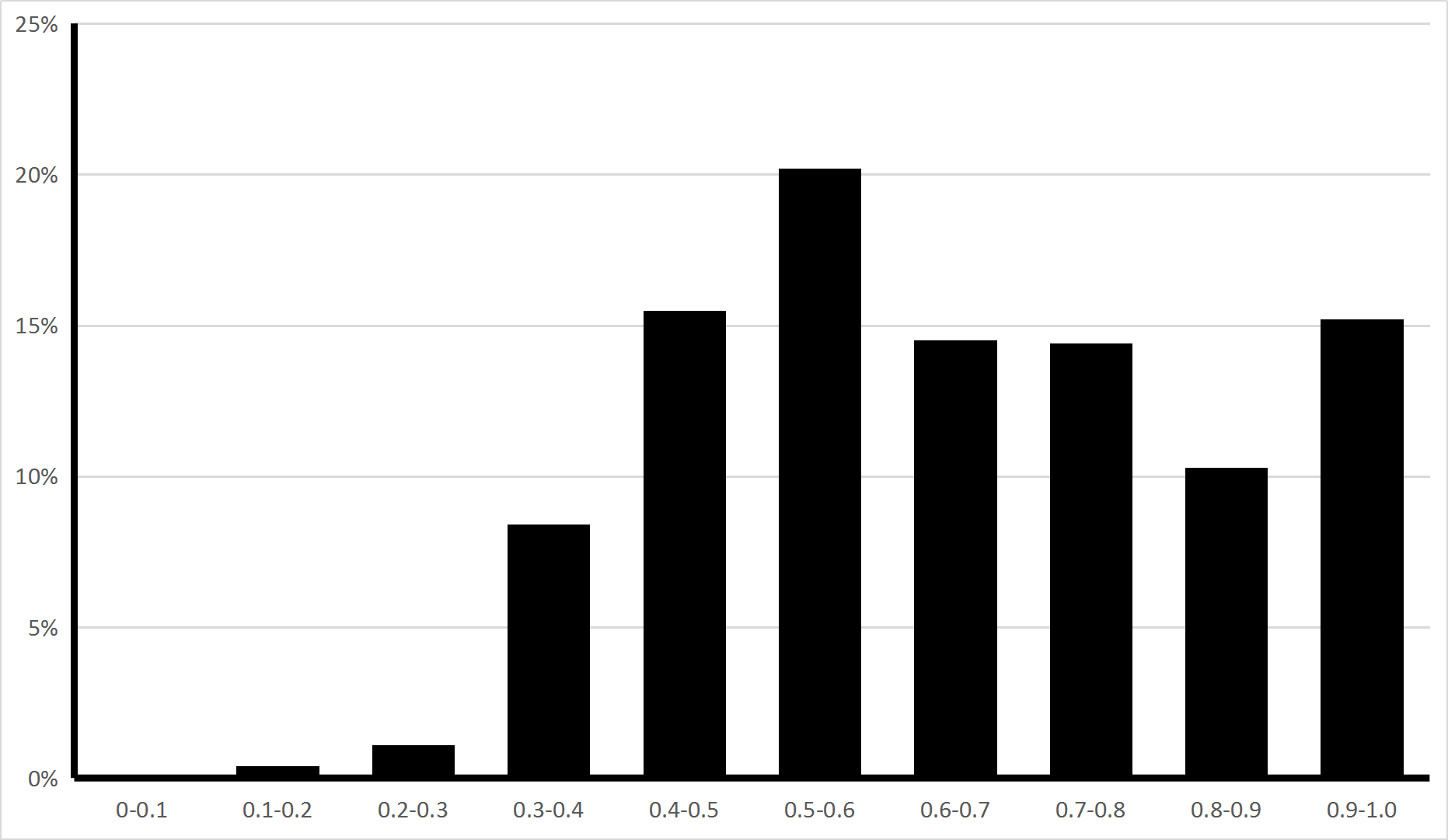

Supplement: FIGURE S1 — Different inherited segments in the Mira genome defined based on diploid single nucleotide polymorphisms (SNPs). [file Image_1.TIF]

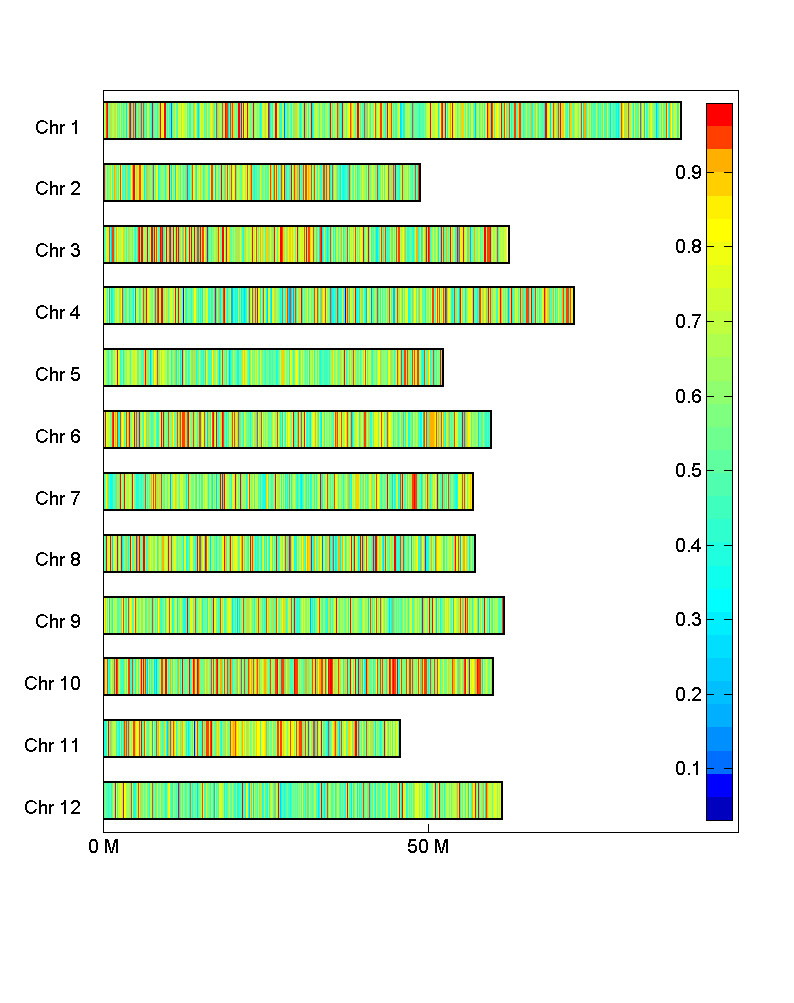

Supplement: FIGURE S2 — Histogram showing the distribution of inherited ratio calculated based on diploid SNPs. [file Image_2.TIF]

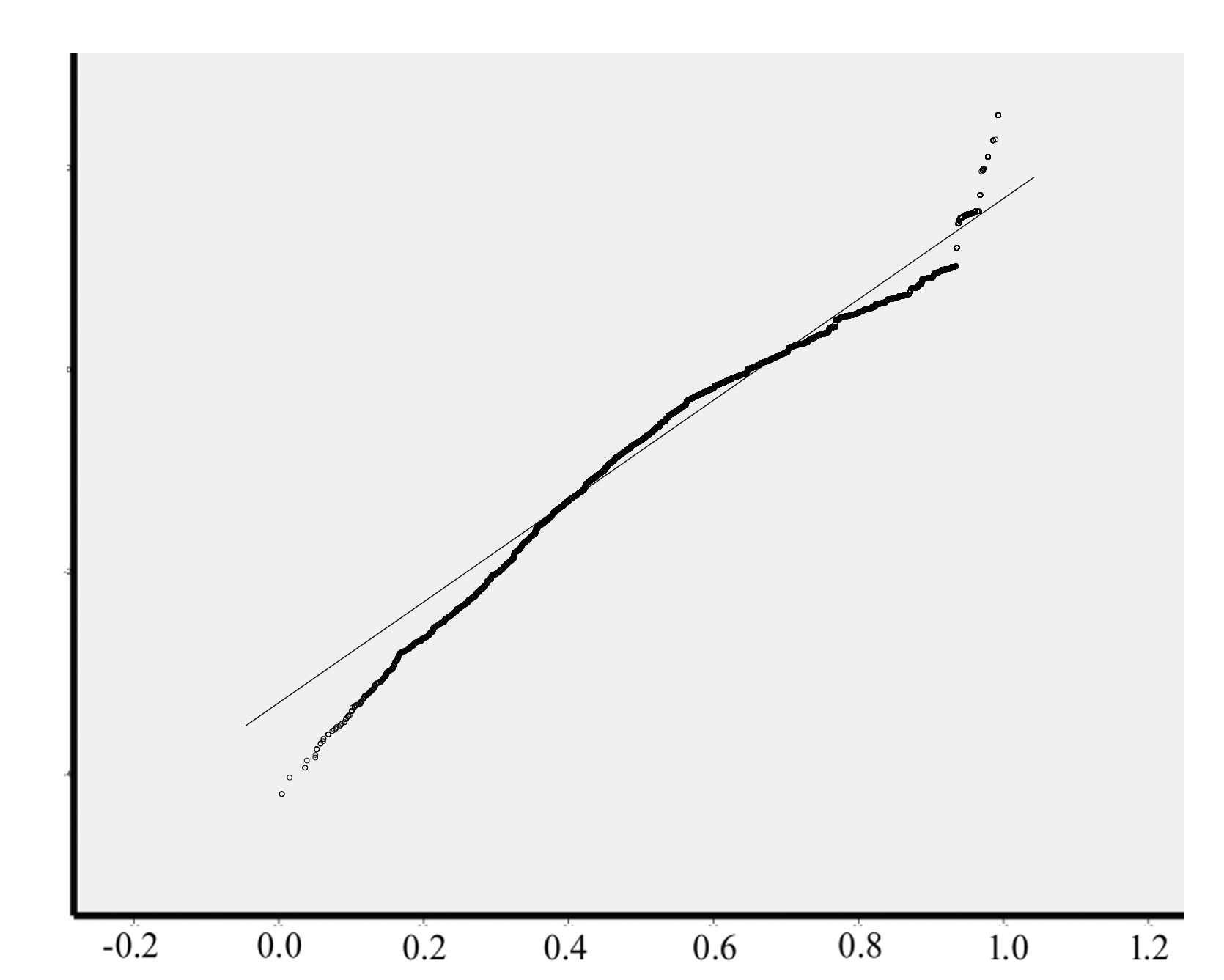

Supplement: FIGURE S3 — QQ plot showing the distribution of inherited ratio calculated based on diploid SNPs. [file Image_3.TIF]

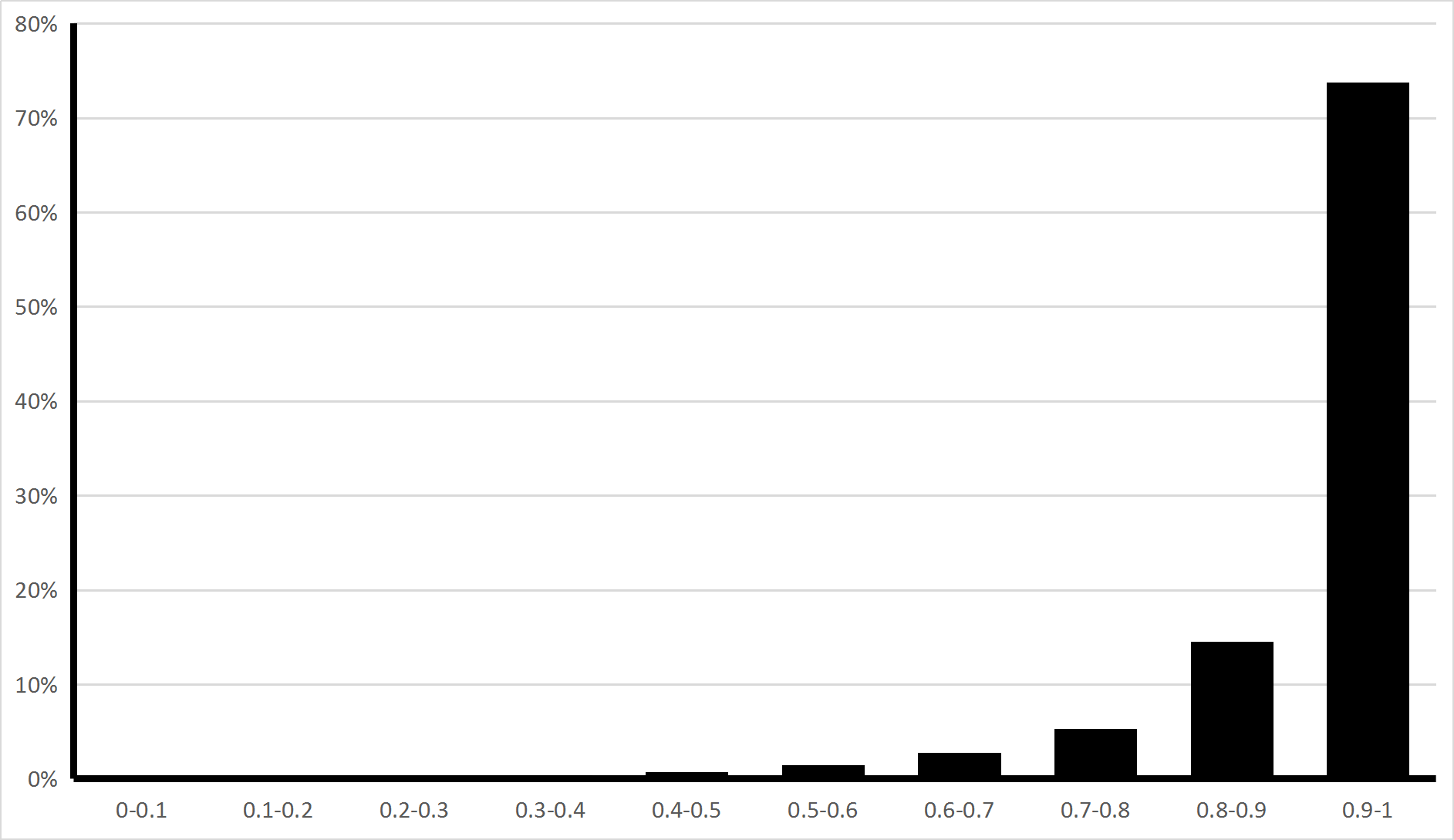

Supplement: FIGURE S4 — Histogram showing the distribution of inherited ratio calculated assuming all progenies have the same pedigree status. [file Image_4.TIF]

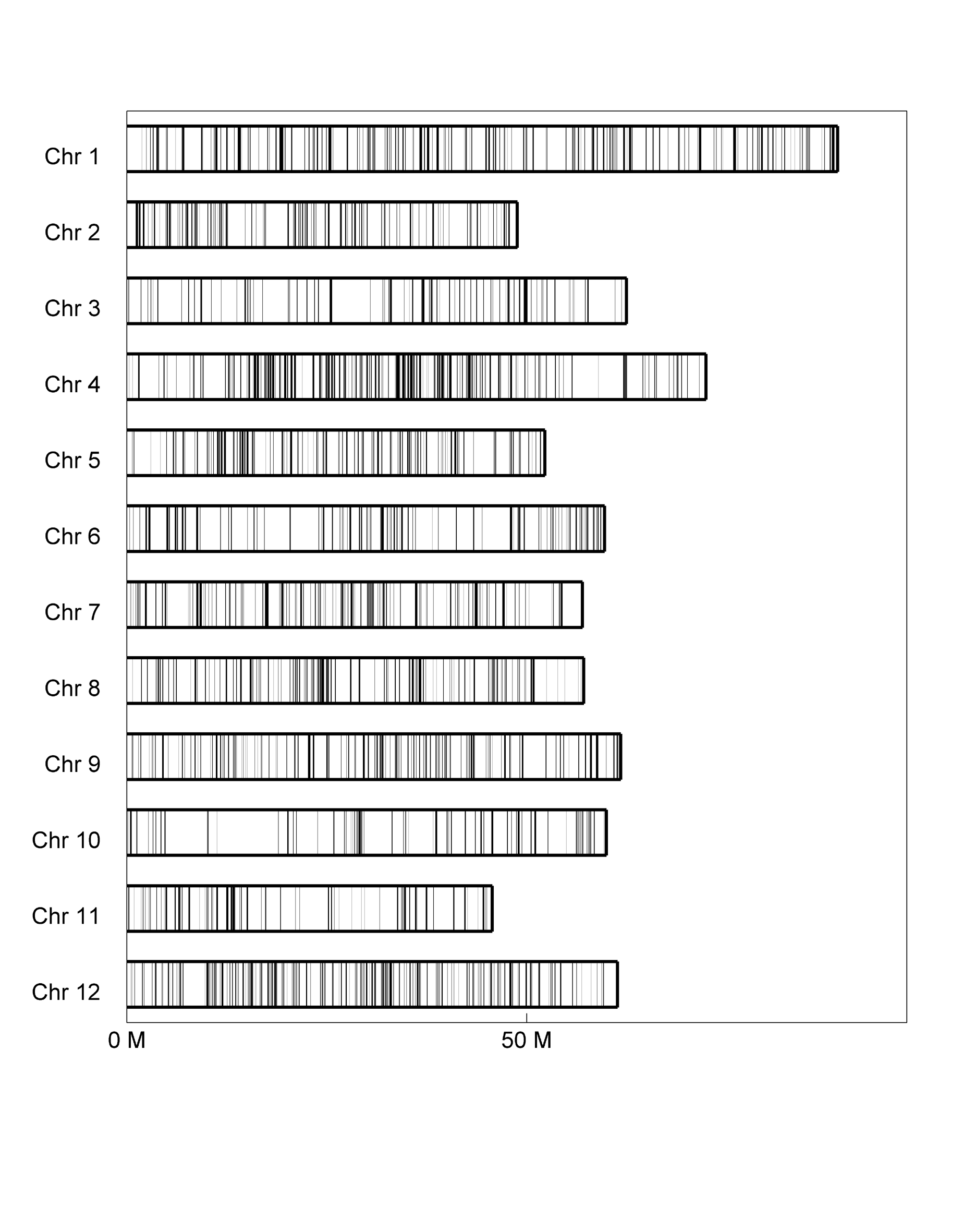

Supplement: FIGURE S5 — Distribution of highly variable segments. [file Image_5.TIF]
